# Low-Cost Indoor Positioning Application Based on Map Assistance and Mobile Phone Sensors

**DOI:** 10.3390/s18124285

**Published:** 2018-12-05

**Authors:** Yi-Shan Li, Fang-Shii Ning

**Affiliations:** 1 Department of Environmental Information and Engineering, Chung Cheng Institute of Technology, National Defense University, Taoyuan 33551, Taiwan; yslee602@gmail.com; 2 Department of Land Economics, National Chengchi University, Taipei 11605, Taiwan

**Keywords:** indoor positioning, mobile phone sensors, pedestrian dead reckoning (PDR), map assistance

## Abstract

Current mainstream navigation and positioning equipment, intended for providing accurate positioning signals, comprise global navigation satellite systems, maps, and geospatial databases. Although global navigation satellite systems have matured and are widespread, they cannot provide effective navigation and positioning services in covered areas or areas lacking strong signals, such as indoor environments. To solve the problem of positioning in environments lacking satellite signals and achieve cost-effective indoor positioning, this study aimed to develop an inexpensive indoor positioning program, in which the positions of users were calculated by pedestrian dead reckoning (PDR) using the built-in accelerometer and gyroscope in a mobile phone. In addition, the corner and linear calibration points were established to correct the positions with the map assistance. Distance, azimuth, and rotation angle detections were conducted for analyzing the indoor positioning results. The results revealed that the closure accuracy of the PDR positioning was enhanced by more than 90% with a root mean square error of 0.6 m after calibration. Ninety-four percent of the corrected PDR positioning results exhibited errors of <1 m, revealing a desk-level positioning accuracy. Accordingly, this study successfully combined mobile phone sensors with map assistance for improving indoor positioning accuracy.

## 1. Introduction

Global navigation satellite systems (GNSS) are used for a broad range of purposes in our daily life, such as rescue positioning, route navigation, traffic monitoring, and agricultural production. However, GNSS have limitations, such as disturbances in positioning signal stability in indoors because of their obstacle environments. This has prompted the development of indoor positioning systems.

A lack of GNSS signals poses a major challenge in capturing indoor spatial information. Numerous approaches have been designed for indoor positioning, such as the early Active Bat, that utilizes ultrasonic technology and simultaneous localization and mapping, which is applied in conjunction with self-propelled robot movement. Most of these approaches involve wireless technologies, such as Bluetooth, infrared, radio frequency identification, wireless fidelity, Zigbee, and ultra-wideband. Bluetooth is a short-range wireless technology for transferring data between different electronic devices based on the IEEE 802.15 physical layer (PHY) specifications. Infrared (IR) wireless is the use of wireless technology for data transferring through a line-of-sight (LOS) IR radiation communication mode based on the IEEE 802.11 PHY specifications. Radio-frequency identification (RFID) is a radio wave technology for transferring data to identify and track tags attached to objects based on the IEEE 802.15 PHY specifications. Wireless fidelity (Wi-Fi) is a radio technology for local area networking of devices based on the IEEE 802.11 PHY specifications. Zigbee is a low-power wireless technology for personal area networking based on IEEE 802.15.4. Ultra-wideband (UWB) is a short-range and high-bandwidth radio wireless technology for networking based on the IEEE 802.15.4a. The comparison of these wireless technology for indoor positioning as shown in Table 1 [1,2,3,4,5,6,7,8,9,10,11,12,13].

Although the wireless indoor positioning technologies mentioned in Table 1 can reach positioning accuracy ranging from centimeters to meters, these technologies are still difficult to use with certain conditions, such as the purchase and arrangement of wireless devices. Recently, the evolution of microelectromechanical systems (MEMSs) and the miniaturization of electronics enable the mass production of wafer physical sensors for detecting angular velocity and acceleration [14]. These wafers are small in volume, lightweight, and energy-efficient, and they have been applied in automotives, indoor positioning and navigation, and other industrial applications. Therefore, MEMSs can be used to develop small, inexpensive, and energy-efficient navigation systems [15]. Most mobile phones, today, contain inertial measurement unit sensors (IMUs), such as MEMS accelerometers, magnetometers, and gyroscopes. These sensors, which are initially designed for screen orientation (automatic rotation) or pace counting, can also be applied for positioning.

Pedestrian dead reckoning (PDR) is a relative positioning technique that uses IMU data to calculate pedestrian location. Compared with other wireless positioning systems, PDR is easy to operate and low-cost for common users, because no additional equipment is needed. However, the PDR results estimated from IMU data have accumulated errors with time. PDR positioning error accumulates over time. In this regard, different researchers have proposed different methods for correcting the PDR positioning errors, including correcting by combining other sensors and wireless devices [16,17], and algorithmic advancements for heading direction and step length estimations [18,19,20,21,22,23]. In addition, the concept of calibration points has also been proposed [24,25]. PDR positioning errors over time can be corrected by the calibration points with known coordinates. However, the rules for calibration point settings are not explicitly stated in the above studies. This study focuses on the calibration point setting rules and the PDR correction results to improve the accuracy of PDR positioning. Through the principles of setting calibration points proposed in this study, the PDR indoor positioning based on map assistance can automatically set the appropriate calibration points according to different indoor planes, and achieve high precision and low-cost indoor positioning effects.

## 2. Materials and Methods

**Figure 1:**
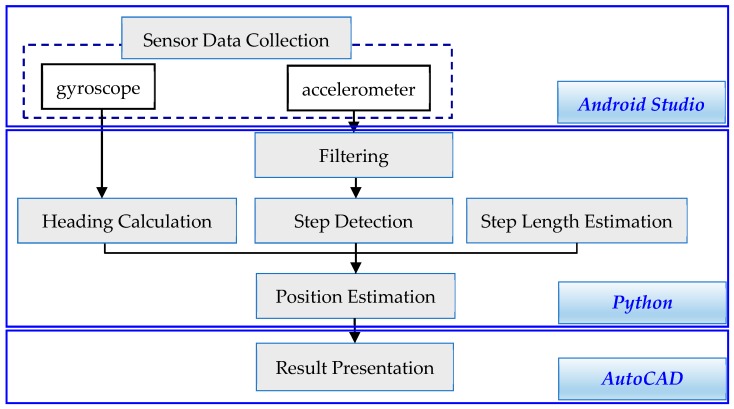
Pedestrian dead reckoning (PDR) structure.

According to the 3-month data published by Kantar (a mobile phone market research company) in December 2017, Android is the mobile phone operating system with the highest market share [26]. In a comparison of the data with the 3-month data from December 2016, Android saw a rise in its market share in most countries [26]. Therefore, this study used an Android phone, HTC One (M8), as the primary agent in the experiment. In this study, two kinds of sensors, a gyroscope and accelerometer, were used. The two-sensor data could be used for heading calculation and step detection, respectively (Figure 1). The results for HTC One (M8) were a theoretical experiment about the PDR results discussion. There were different drifts and offsets in different mobile phone sensors. It was necessary to obtain the error constant of the sensors in different mobile phone models through experiments. However, the positioning principle was consistent, and the relevant methods proposed in this study should be applicable.

Table 2 lists the specifications of the experimental mobile phone. In this study, Android Studio was used for test environment development and data export from sensors. Sensor data were transferred to a computer via mobile entity or email for calculation. Then, Python was employed for algorithm compilation system integration through interfacing with AutoCAD, thereby illustrating the results. In other words, sensor data collection and PDR algorithm compilation were worked by Android Studio and Python, respectively. Through interfacing by Python, the final PDR positioning results were automatically mapped and presented by AutoCAD (Figure 1).

### 2.1. Pedestrian Dead Reckoning

PDR, which is a relative positioning technology, estimates users’ outdoor or indoor positions through the measurement of moving distances and directions from the users’ initial positions with inertial sensors, which are expressed as follows:

(1) $$X_{t}=X_{t-1}+{\hat{s}}_{\left[t-1,t\right]}\sin{\hat{\theta }}_{\left[t-1,\;t\right]},$$

(2) $$Y_{t}=Y_{t-1}+{\hat{s}}_{\left[t-1,t\right]}\cos{\hat{\theta }}_{\left[t-1,\;t\right]},$$

where $\left(X_{t},\;Y_{t}\right)\;\mathrm{and}\;\left(X_{t-1},\;Y_{t-1}\right)$ represent the coordinates at the time *t* and (*t* − 1), respectively; ${\hat{s}}_{\left[t-1,t\right]}$ represents the moving distance from (*t* − 1) to t, which was defined as the length of a user’s step in this study; and ${\hat{\theta }}_{\left[t-1,\;t\right]}$ indicates the user’s moving direction at (*t* − 1).

PDR typically comprises three phases, namely, heading calculation, step detection, and step length estimation (Figure 1), all of which are detailed in this subsection.

#### 2.1.1. Heading Calculation

Heading refers to the moving direction of the device and its user. To simplify the test data, the user was asked to affix the device to their chest for heading consistency. In the present study, the heading referred to the rotation angle during the user’s movement. The known map data were used to determine the initial angle of movement as the starting rule for the gyroscope in its calculation.

Heading calculation typically involves using gyroscope and magnetometer data or the combined algorithm of the two. This study only adopted the gyroscope data because of the phone’s specifications. The data were employed in calculating the heading angle during the user’s movement, which was applied jointly with step detection results for calculating the user’s coordinates. The equation of the heading angle is

(3) $$Ori_{t}=\;Ori_{t-1}+{\hat{\theta }}_{\left[t-1,\;t\right]},$$

where $Ori_{t}$ and $Ori_{t-1}$ represent the heading angles at *t* and (*t* − 1), respectively, and ${\hat{\theta }}_{\left[t-1,\;t\right]}$ represents the rotation angle from (*t* − 1) to *t*.

**Figure 2:**
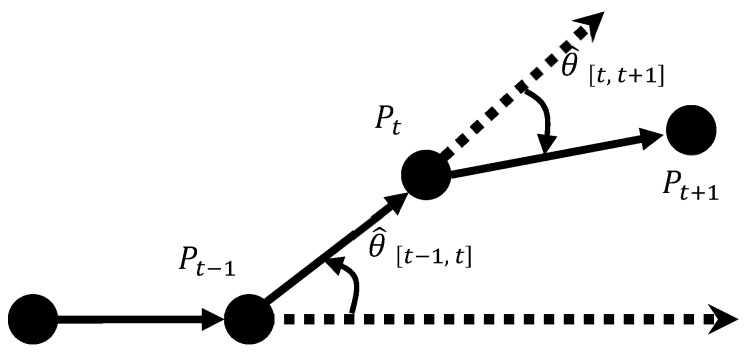
Diagram of heading angle.

Figure 2 illustrates the calculation of the heading angle, where the solid lines refer to the movement paths; the dotted lines refer to the tangent directions of the preceding paths; $P_{t-1}$, $P_{t}$ and $P_{t+1}$ represent the positions of *P* at (*t* − 1), *t*, and (*t* + 1), respectively; ${\hat{\theta }}_{\left[t-1,\;t\right]}$ and ${\hat{\theta }}_{\left[t,\;t+1\right]}$ represent the rotation angles from (*t* − 1) to *t* and from *t* to (*t* + 1), respectively; and the heading angle from (*t* − 1) to (*t* + 1) can be expressed as $Ori_{\left[t-1,\;t+1\right]}={\hat{\theta }}_{\left[t-1,\;t\right]}+{\hat{\theta }}_{\left[t,\;t+1\right]}$. Moreover, rotation angle $\hat{\theta }$ is defined as positive in counterclockwise rotation, and negative in clockwise rotation.

The gyroscope was used to detect the angular velocity (ω), which refers to the changes of the angle dθ over the time *dt*. That is,

(4) $$\omega \;=\;\frac{d\theta }{dt}.$$

**Figure 3:**
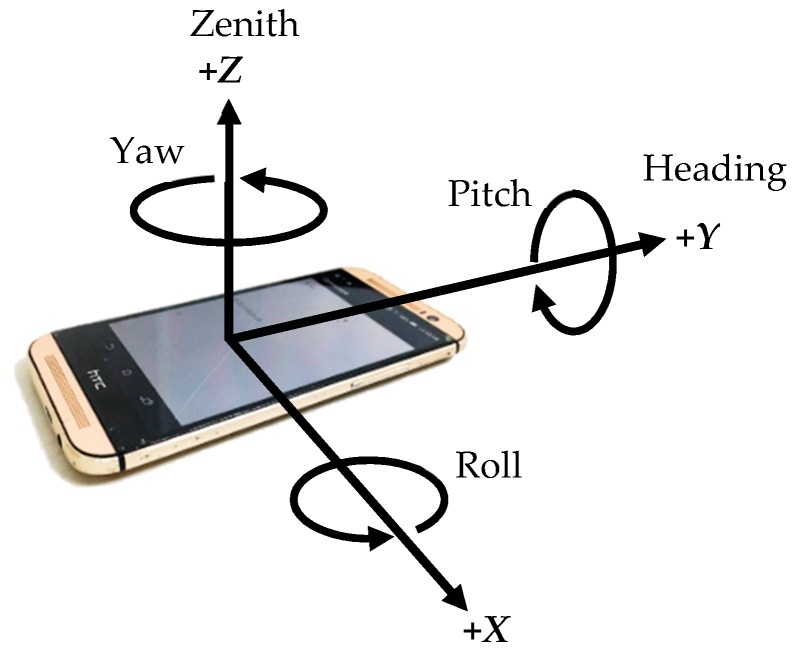
Axial directions of the mobile phone gyroscope.

Angular velocity is typically expressed in degrees per second (dps, °/s). Figure 3 illustrates the three axial directions of the gyroscope, where *z*-axis refers to the zenith direction, *Y*-axis refers to the heading direction, and *X*-axis forms a right-handed coordinate system that is orthogonal to the other axes. The angle was the first-order integral of the angular velocity, and the concurrent angle changes were identified through the integration of the sampling time and angular velocity data [24]. The velocity was designated as zero at the initial heading direction (straight ahead), positive in counterclockwise rotations, and negative in clockwise rotations. Since the PDR system required precise data on the angle changes in the sensor, smoothing the sensor signals through filters might have caused the incapacity of the angle calculation results to represent the real movement. Therefore, the gyroscope data in this study were not filtered [25].

#### 2.1.2. Step Detection and Step Length Estimation

Steps have mostly been detected through the GNSS measurement of the movement distance and the subsequent reverse inference of the number of steps. Although effective, this approach is inapplicable in indoor environments, or in devices without GNSS signals. Indoor steps can be detected through accelerometers, which can work satisfactorily both in devices that are incompatible with a global positioning system and in conjunction with a GNSS. Numerous algorithms applicable for step detection in accelerometers are available, such as the zero-crossing method [27,28], autocorrelation [29,30,31], and peak detection [32,33,34,35,36]. This study adopted the peak detection approach for step detection through an accelerometer, in which the maximal acceleration during the user’s movement was used for calculating the steps.

**Figure 4:**
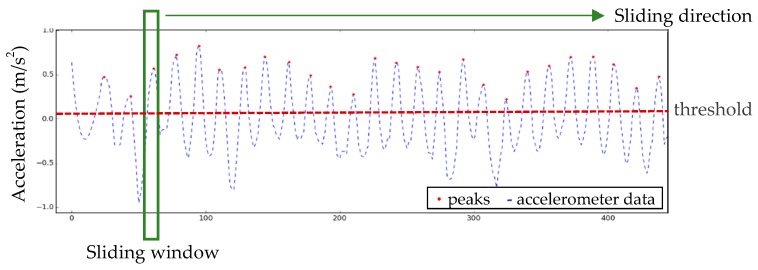
Diagram of peak detection.

In the peak detection approach, steps can be detected on the basis of the gait cycle. The accelerometer detected the changes in the step intervals corresponding to the gait cycle to identify the steps of the user. Since the signal eigenvalue values appeared within a specific range, applying the sliding-window algorithm could enhance the step detection results [24]. This study adopted seven sliding-window sizes, and designated the threshold value of the peak detection as 0.0005 m/s^2^ (Figure 4). 

The accelerometer data were processed through the use of the Savitzky–Golay (SG) filter; noises were eliminated before the pace detection commenced. The SG filter is a local least-squares polynomial data-smoothing method proposed by Savitzky and Golay [37]. This method involves applying a least-squares polynomial regression to replace the original data with the weighted means of their adjacent points, thereby smoothing the data and reducing the noises. Thus, the precision of the prediction model is enhanced, and the prediction errors are reduced. Additionally, the shape and width of the signal remain unchanged, while the shapes and intensity of the waveform peaks are maintained [37,38,39]. Accordingly, the SG filter is applicable for processing data from indoor positioning sensors, and was applied in the present study.

**Figure 5:**
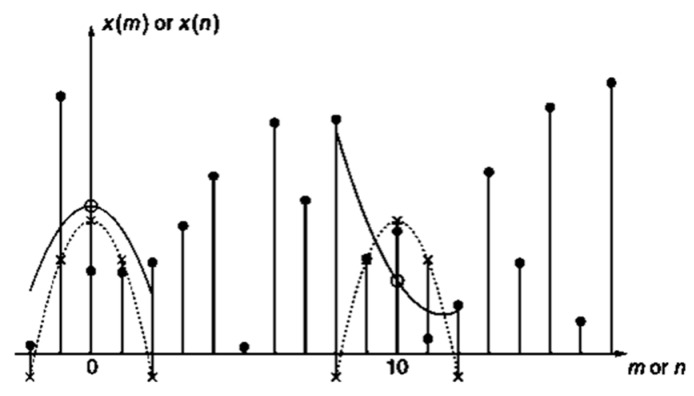
Diagram of the least-squares polynomial fit in the Savitzky–Golay (SG) filter [39].

Figure 5 shows the basic concept of the SG filter, where solid circle symbol (●) refers to the samples under observation, hollow circle symbol (○) represents the output samples based on the least squares, and cross symbol (×) represents the impulse response samples and the weight constant. The dotted curve represents the polynomial approximates of the central unit impulse, and the solid curve represents the locally fitted quadratic polynomial. 

When a series of data *x* [*n*] is given, a group of 2*M* + 1 data, centered on *n* = 0, are considered and are fitted through

(5) $$p\left(n\right)=\sum_{k=0}^{N}a_{k}n^{k},$$

where the residual of the least-squares fit is

(6) $$\varepsilon_{N}=\sum_{n=-M}^{M}{\left(p\left(n\right)-x\left[n\right]\right)}^{2}=\sum_{n=-M}^{M}{\left(\sum_{k=0}^{N}a_{k}n^{k}-x\left[n\right]\right)}^{2},$$

where *M* represents the half value of the estimation interval. Figure 5 illustrates the estimation interval of 2*M* + 1. The solid curve on the left represents the fitting results when *M* = 2 and *N* = 2. Regarding the fitting sequence with 2*M* + 1 as the estimation interval, the center point of the interval shifts from *n* = 0 to *n* = 1 when a fitting phase is over, and another polynomial fit commences. Thus, all the input samples undergo the fitting process.

The fitting result when *n* = 0 is

(7) $$y\left[0\right]=p\left[0\right]=a_{0}.$$

Only the constant term of the polynomial was required to complete the fitting process. Subsequently, the weighted averages of the samples were calculated through the convolution theorem. That is,

(8) $$y\left[n\right]=\sum_{m=-M}^{M}h\left[m\right]x\left[n-m\right]=\sum_{m=n-M}^{n+M}h\left[n-m\right]x\left[m\right],$$

where *h*[*m*] is a finite impulse response value, which was adopted as a weighted value. Regarding *a*_0_, according to the least-squares principle, when the fit residual $\varepsilon_{N}$ represents a minimal value, its partial differential with each parameter should be zero. That is,

(9) $$\frac{\partial \varepsilon_{N}}{\partial a_{i}}=\overset{M}{\underset{m=-M}{\sum }}2n^{i}\left(p\left(n\right)-x\left[n\right]\right)=\overset{M}{\underset{m=-M}{\sum }}2n^{i}\left(\overset{N}{\underset{k=0}{\sum }}a_{k}n^{k}-x\left[n\right]\right).$$

It can be reorganized as

(10) $$\sum_{k=0}^{N}\left(\sum_{n=-M}^{M}n^{i+k}\right)a_{k}=\sum_{n=-M}^{M}n^{i}x\left[n\right],i=0,1,\ldots ,N.$$

A design matrix **A** was applied in the follow solution process. That is,

(11) $$A_{\left(2M+1\right)\times \left(N+1\right)}=\left[\begin{array}{ccccccc} a_{-M,0} & a_{-M+1,0} & \ldots & a_{0,0} & \ldots & a_{M-1,0} & a_{M,0}\\ a_{-M,1} & a_{-M+1,1} & \ldots & a_{0,1} & \ldots & a_{M-1,1} & a_{M,1}\\ \vdots & \vdots & \vdots & \vdots & \vdots & \vdots & \vdots \\ a_{-M,N} & a_{-M+1,N} & \ldots & a_{0,N} & \ldots & a_{M-1,N} & a_{M,N} \end{array}\right],$$

where $a_{n,i}=n^{i},\;-M\leq n\leq M,\;0\leq n\leq N$. The auxiliary matrix **B** is applied, and **B** = **A**^T^**A**, which is calculated by

(12) $$b_{i,k}=\sum_{n=-M}^{M}a_{i,n}a_{n,k}=\sum_{n=-M}^{M}n^{i+k}=b_{k,i}.$$

The data entered are defined as the matrix **x**, and the undetermined coefficients are defined as the matrix **a**. They are

(13) $$x=\left[\begin{array}{c} x\left[-M\right]\\ \vdots \\ X\left[M\right] \end{array}\right],\;a=\left[\begin{array}{c} a\left[0\right]\\ \vdots \\ a\left[N\right] \end{array}\right].$$

The matrix **a** is calculated according to **B** = **A**^T^**A**. They are

**Ba** = **A**^T^**A*a*** = **A**^T^**x**, (14)

**a** = (**A**^T^**A**)^−1^**A**^T^**x** = **Hx**, (15)

where the first row of the matrix **H** is the undetermined convolution coefficient. For a detailed verification of the aforementioned equations, refer to the findings in [37,39].

In this study, map assistance employed the known indoor map for positioning, and the movement distance could be acquired through the map. The distance on the map was divided by the step detection results above, to determine the movement distance in each linear path. The simplified step length equation was

(16) SL=D/P,

where SL refers to step length, *D* represents the movement distance, and *P* represents the number of steps, the result of which was employed for the follow-up position calculation.

### 2.2. Establishing a Basic Map

Modern people spend more than 70% of their time indoors, preventing standard GNSS from providing services [40]. Therefore, accurate indoor positioning technology plays a key role in providing applicable and targeted positioning service to indoor users. Indoor maps are an indispensable part of indoor positioning systems. The current acceptable range of positioning accuracy in the existing indoor positioning technology is 5–10 m, which is a room-level positioning accuracy. Reducing the range to 1–2 m (a desk-level accuracy) is desirable, for enhanced effectiveness in position signal transmission [13].

This study adopted the commercial sensor within mobile phones to capture positioning data. The sensor was relatively low-cost, but exhibited certain risks of errors. For example, the inertial measurement unit-based positioning leads to signal drifts over time, which undermines the reliability of the positioning service. Therefore, error calibration must be considered while designing the indoor positioning system to yield more accurate positioning results.

**Figure 6:**
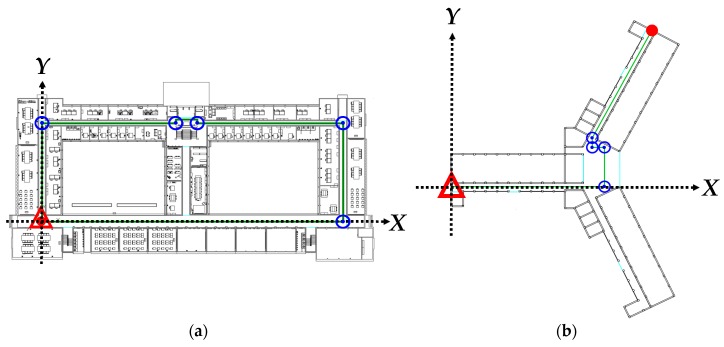
Paths and corner calibration points in the experimental fields: (**a**) Field 1; (**b**) Field 2. (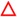 start point; 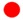 end point; 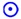 calibration point on the corner; 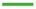 true path.)

To keep the positioning results within the reasonable range of errors, known indoor floor plan were employed for positioning constraints. The first floors of two unique buildings were designated as the experimental fields, labeled as Fields 1 and 2, and were illustrated as maps by using AutoCAD (Figure 6). 

The lower left corner in each field was established as the starting point of the planned route with relative coordinates of (0, 0), and the areas to the right of the *X*-axis and above the *Y*-axis yielded positive values. The user was assumed to walk along the centerline of the aisle in both fields in the indoor positioning test and data collection. The walking path in Field 1 was a closed path, where the starting and ending points were at the same spot, and the intervals between the turns were not too small. The walking path in Field 2 was a connecting path, and the intervals between the turns were particularly small. Table 3 presents the basic details of the positioning tests conducted in the two fields. 

### 2.3. PDR Correction

Due to the prevalence of propagation error or irregular drifts in most indoor positioning technologies, this study implemented known indoor maps for positioning calibration, thereby reducing the effect of system errors on the positioning results. In addition to applying the PDR algorithm, the calibration points on the linear paths and corners were implemented. By employing the distance, azimuth, and rotation angle detections, the accuracy of PDR positioning could be improved.

#### 2.3.1. Establishing the Calibration Points

For consistency between the PDR positioning results and the actual walking paths, two types of calibration points, namely corner and linear, were established for PDR path calibration. The corner calibration points were established at the corners of the paths (Figure 6). In addition, linear calibration points were established along the linear paths in accordance with their basic specifications, which were determined according to the gyroscope drifts.

**Figure 7:**
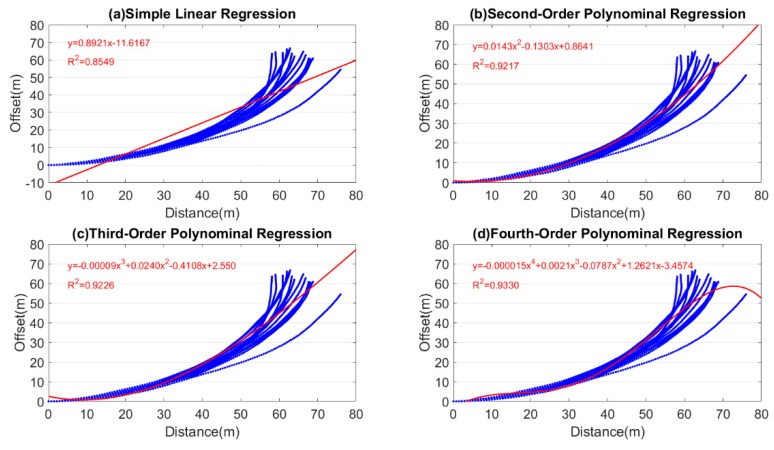
Regression analysis of the linear experimental field.

**Figure 8:**
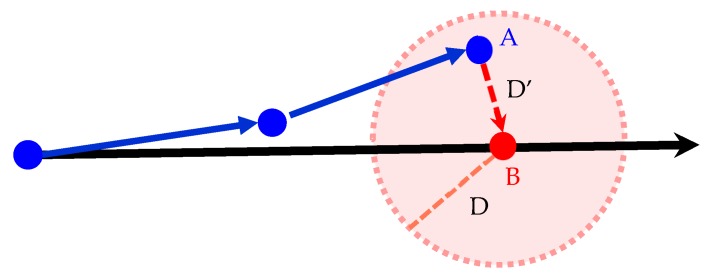
Diagram of distance detection. (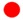 calibration point; 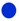 sensor point; 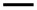 true path; 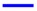 sensor path.)

Regression analysis was conducted to calculate the degree of drifts in the gyroscope to establish the specifications for the linear calibration points. The user was asked to walk on a 90 m linear testing field 20 times, and the associated data underwent regression analysis according to the PDR-calculated relative coordinates. Figure 7 shows the results of the simple linear, quadratic polynomial, cubic polynomial, and quartic polynomial regression analyses. Figure 7a illustrates the simple linear regression analysis ($R^{2}=0.8549$), Figure 7b the quadratic polynomial regression analysis ($R^{2}=0.9217$), Figure 8c the cubic polynomial regression analysis ($R^{2}=0.9226$), and Figure 8d the quartic polynomial regression analysis ($R^{2}=0.9330$). The *X*-axis values represent the linear walking distance, and the *Y*-axis value represents the amount of drift. As shown in Figure 7a, the simple linear regression results were significantly different from the actual data distribution, and exhibited a low coefficient of determination (*R*^2^). Therefore, this analysis approach was not adopted. Conversely, the quadratic, cubic, and quartic polynomial regression results exhibited nonsignificant differences in *R*^2^. However, the quartic polynomial regression equation was susceptible to the gross error in the observation data. Consequently, after the walking distance reached 60 m, extreme values appeared in the regression function, leading to data overfitting. Therefore, the quartic polynomial regression was not adopted in this study. Due of the similar results from the quadratic and cubic polynomial regressions, the quadratic polynomial with lower order was adopted for regression analysis under the consideration of a simple model with fast and convenient calculation. The equation of this regression model is $Y=0.0001X^{2}-0.1303X+86.413$ (Figure 7b).

In the quadratic polynomial regression equation, *Y* represents the amount of path drift and X represents the linear walking distance. Since the user was assumed to walk along the centerline of the aisle, the threshold value of the drift (*Y* value) was set as half the minimal width of the aisle (see Table 3 for the minimal width). According to the quadratic polynomial regression model, the intervals between each pair of linear calibration points (*X* value) were 12 m in Field 1, and 13 m in Field 2. Equidistant linear calibration points were established according to the interval calculation results, and the intervals were adjusted automatically as integer multiples of step distances, and interlocked with the distances through programming.

#### 2.3.2. Distance Detection

Through the detection of the distances between the sensor point and calibration point, the sensor points that satisfied the conditions were distributed to the nodes closest to the geometric distances of the sensor points (Figure 8). The length of D’ is the distance between Calibration Point B and Sensor Point A, and the length of D is the threshold value of set distance. The calibration range is the circle with a calibration point as the center and the length D as the radius. When D’ < D, the conditions for correction are satisfied. Meanwhile, azimuth detection, which is detailed in the next section, was then conducted to match Point A to Calibration Point B.

#### 2.3.3. Azimuth Detection

**Figure 9:**
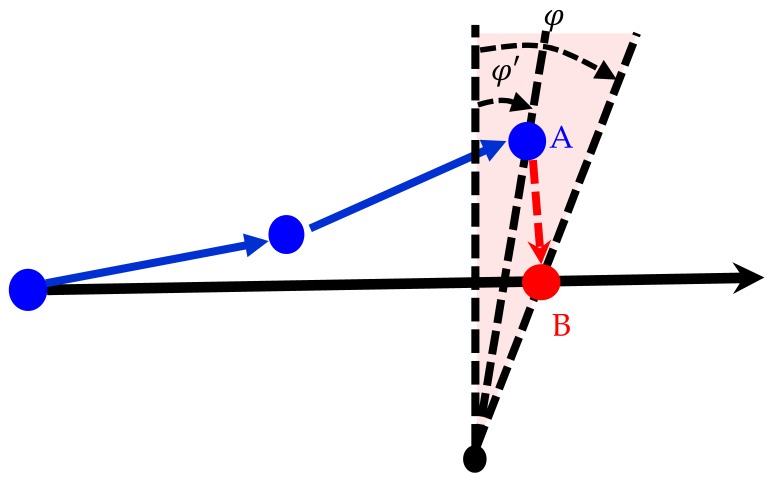
Diagram of azimuth detection. (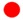 calibration point; 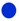 sensor point; 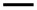 true path; 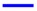 sensor path.)

This detection was performed in conjunction with aforementioned distance detection. A known point from the map was used to calculate the azimuth φ′ from the sensor point and the azimuth φ from the calibration point. The azimuth φ was the threshold of azimuth value. When $\varphi^{\prime }$ < φ, the sensor point had entered the range required for calibration, and azimuth detection was conducted in conjunction with the results of the distance detection to match Point A to Calibration Point B (Figure 9). 

#### 2.3.4. Rotation Angle Detection

**Figure 10:**
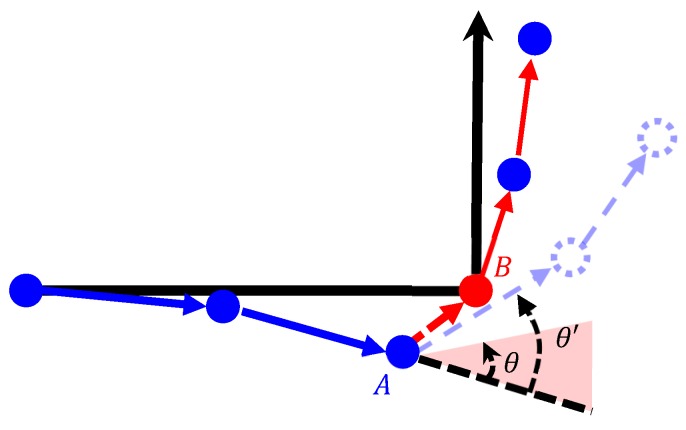
Diagram of rotation angle detection. (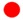 calibration point; 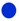 sensor point; 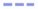 path before calibration; 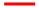 path after calibration.)

**Figure 11:**
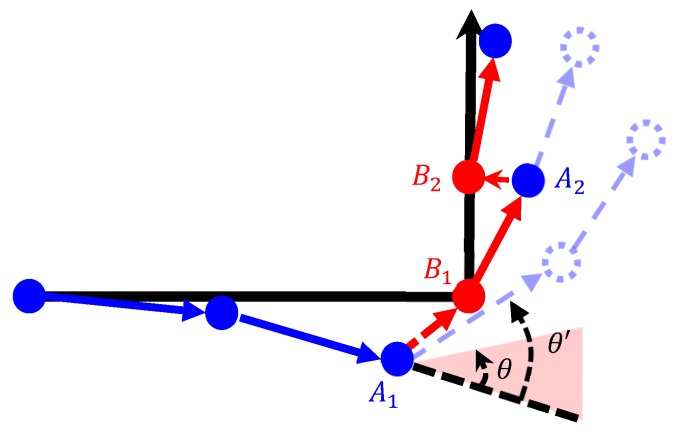
Diagram of calibration after turning. (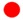 calibration point; 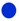 sensor point; 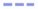 path before calibration; 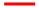 path after calibration.)

Rotation angle detection required the use of gyroscope data for heading observation to detect the changes in the navigation direction, and was mainly employed when the user turned. The threshold value θ of the instantaneous rotation angle was set (θ = 20°), according to the rule of thumb. When the absolute value of $\theta^{\prime }$ exceeded θ, the point of the angle was considered a turning point, and the aforementioned Point A was matched to the turning Calibration Point B (Figure 10). According to the data processing results of multiple runs, the action of turning might have appeared as multiple rotation angle data because the user did not immediately turn to the next direction. To reduce this data error, an additional linear calibration point was set in the linear path directly following the turning point to match the calibration result to the actual movement pattern. Figure 11 illustrates the matching of the Sensor Point A_2_ to the Calibration Point B_2_.

## 3. Results and Discussion

The experimental results were divided into 1. PDR positioning results using only the sensors built-in mobile phone (accelerometers and gyroscopes); 2. PDR correction positioning using length, azimuth and rotation angle detection based on map assistance and mobile phone sensors. Relevant experimental results and discussions are presented in the following subsections.

### 3.1. PDR Positioning

The PDR positioning results were the path outcomes calculated through Equations (1) and (2) using the accelerometer and gyroscope data according to the coordinates of each step. The closure error between the estimated end point and the known point in each path was regarded as the error in each path estimation, and the statistical data of the closure errors were used for analyzing the accuracy of the experiment.

**Figure 12:**
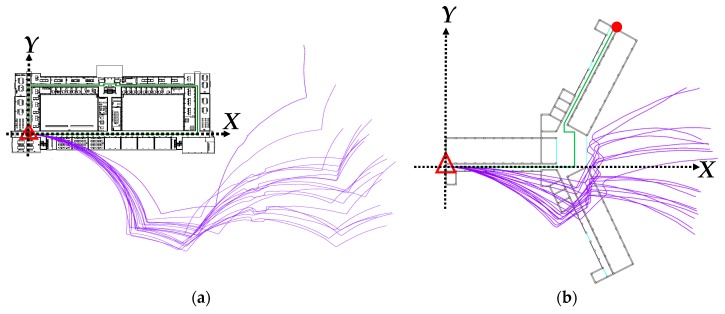
Results of PDR positioning: (**a**) Field 1; (**b**) Field 2. (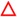 start point; 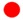 end point; 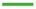 true path; 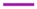 PDR path.)

Twenty sets of movement observation data were recorded along the designed paths in each of the experimental fields in this study, the test outcomes of which are shown in Table 4. The aforementioned closure errors increased as the length of the walking path expanded. The relative accuracy of the closures in the PDR results of the two experimental fields was approximately 1/1.5. Figure 12 illustrates the actual positioning results in both fields, in which the user started from the starting point on the lower left to the indicated ending points. The original PDR-defined paths differed considerably from the actual walking paths; however, special characteristics were still identified at the turning points (e.g., corners and the obstacles on the linear paths).

### 3.2. PDR Correction Positioning

To enhance the accuracy of the PDR positioning results, PDR correction was conducted using the approach in Section 2.3, after which, the observation data in Section 3.1 was adopted for a calibration experiment, thereby improving the PDR positioning results.

**Figure 13:**
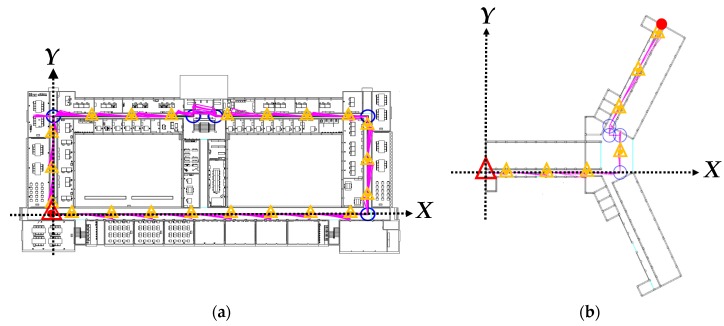
Results of PDR correction: (**a**) Field 1; (**b**) Field 2. (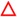 start point; 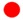 end point; 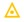 calibration point on the line; 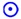 calibration point on the corner; 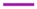 corrected PDR path.)

Table 5 displays the PDR correction results, and Figure 13 shows the corrected paths. Comparing the corrected paths with the PDR results presented in Figure 12 revealed that the corrected paths had a closer resemblance to the designated paths in the experiment. On the closure errors, the positioning errors were reduced to 0.6 m after the correction, revealing that the control of the errors within half the widths of the aisles was successful. The positioning accuracy improved up to 95% after the correction.

**Figure 14:**
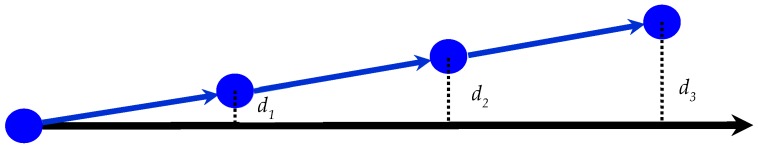
Diagram of vertical offset. (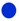 sensor point; 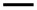 true path; 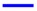 sensor path.)

To further clarify the changes in the positioning errors after calibration, the cumulative passing rate of the errors at the sensor point was calculated. The passing rate, which was the proportion of the positioning errors that matched the number of data points within the threshold value to the total number of positioning points, is expressed as

(17) $$\mathrm{Passing}\;\mathrm{rate}\;\left(\%\right)=\frac{\mathrm{Number}\;\mathrm{of}\;\mathrm{passes}}{\mathrm{Total}\;\mathrm{number}\;\mathrm{of}\;\mathrm{points}}\times 100,$$

where the number of passes represents the point number of errors that fall within the thresholds. The errors were the vertical offsets of the sensor points of the observation path from the actual path, as shown in $d_{1}$, $d_{2}$, and $d_{3}$ of Figure 14.

**Figure 15:**
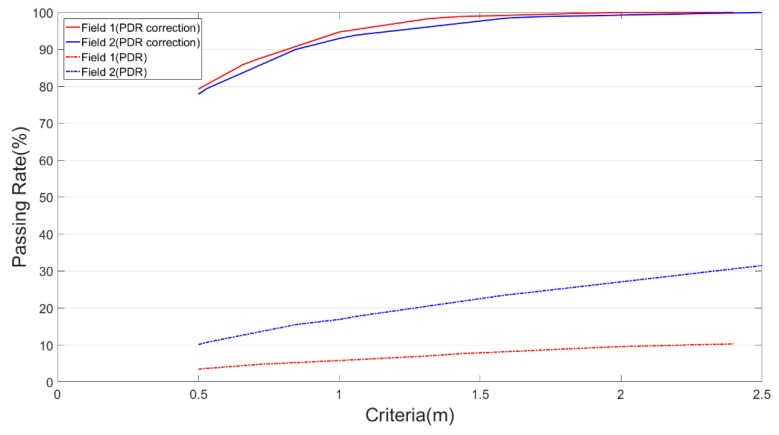
Criteria of vertical offsets.

Table 6 and Figure 15 present the passing rates of the original and corrected PDR values in both experimental fields according to various error thresholds, which revealed a gradual increase in the errors as the walking progressed. Each of the two fields contained 20 sets of test data, Fields 1 and 2 comprised 6250 and 2421 individual data, respectively.

The corrected closure error in the PDR results (different accuracy values were adopted for each experiment field), half the minimal aisle widths (in which the two fields also differed), and the 1 m desk level indoor positioning error were individually used as the error thresholds. Accordingly, the passing rates of the Field 1 PDR correction values at those thresholds were approximately 86%, 87%, and 95%, respectively. The original PDR (before calibration) observation data were examined using the same threshold values, and the passing rates of the positioning errors were revealed to be only 4%, 5%, and 6%, respectively. The PDR correction values reached a 100% passing rate at a threshold value of 2.4 m; however, the original PDR values only reached 10%. Table 6 lists the statistical results of passing rates of the Field 1 positioning results at various error thresholds.

The passing rates of the Field 2 PDR correction values at the aforementioned three error thresholds were 79%, 90%, and 93%, respectively, but those of the non-corrected PDR values were only 11%, 15%, and 17%, respectively. The PDR correction values reached a 100% passing rate at an error threshold of 2.5 m; however, the original PDR values only reached 31%. Table 6 listed the passing rates of the Field 2 positioning results at various error thresholds. Due to the difference between the two fields in the lengths of their walking paths, the PDR results of Field 2 significantly outperformed those of Field 1 in their passing rates by only PDR without correction (dot lines in Figure 15). The test path of Field 1 was 2.7 times as long as that of Field 2. Due to propagation errors and irregular drifts that started to appear in the PDR positioning signals following the increase in the walking distance, the passing rate of the positioning errors in the Field 1 data was obviously lower than that of the Field 2 data. However, both fields were nearly consistent in the passing rates of their corrected PDR data (solid lines in Figure 15), and the errors and drifts in the positioning signals could be controlled within the error thresholds. The corrected PDR signals in Field 1 were slightly more accurate than those in Field 2. This is because of the multiple sharp turns with short intervals, in between, in the middle path section of Field 2, and low sensor accuracy or sensitivity would lead to high positioning errors in these locations.

In summary, according to the analysis of both the closure accuracy and error passing rates, the accuracy of the PDR positioning results improved significantly after calibration. The effectiveness of the improvement increased with the walking distance, and the resemblance of the sensor performance in tracking the user’s movement to the actual movement patterns was enhanced.

## 4. Conclusions

Since GNSS signals are covered and contain multiple paths, maintaining accurate navigation results in closed environments, such as indoor facilities, buildings, and forests, is difficult. This study applied a personal smart mobile device in PDR navigation and implemented known indoor maps for calibrating and improving indoor positioning. Through the principles of setting calibration points proposed in this study, the PDR indoor positioning based on map assistance can automatically set the appropriate calibration points according to different indoor planes and achieve high precision and low-cost indoor positioning effect. The conclusions are as follows:

1. The built-in sensors in the phone and PDR acquired the basic number of steps and navigation data for calculating positions, but the estimation results produced major errors due to propagation errors and low sensor accuracy. The paths calculated through the sensors alone differed significantly from the actual paths, and the relative closure accuracy was only 1/1.5. Therefore, calibration conditions must be applied to PDR for subsequent positioning calculation.
2. Known indoor maps were successfully implemented for setting the calibration points, which were divided into corner and linear calibration points. Thresholds and conditions were established according to the characteristics of these points, which assisted in calibrating the PDR positioning results and improving their resemblance to the actual paths.
3. Regression analysis was successfully performed to calculate the minimal layout intervals between linear calibration points, thereby minimizing the number of control points required in indoor positioning. In addition, this study proved that linear or other regressions were not the most suitable for mobile phone sensor data, but quadratic regression.
4. Establishing calibration points according to known map information was verified to enhance the closeness of the PDR positioning results to the actual paths. The accuracy of the PDR results improved by 95%, and exhibited a root mean square error of 0.6 m after calibration. Moreover, 94% of the calibrated data exhibited errors of <1 m, revealing a desk-level positioning accuracy.

Minimizing the costs and technological threshold, which were achieved through the adoption of software and hardware that are openly authorized and easily accessible, were the top priorities in the indoor positioning program in this study. Therefore, a mobile device with built-in sensors is adopted for PDR positioning, and freeware programs, namely Android Studio and Python, are employed for writing the program. Thus, developing a highly accurate indoor positioning system without expensive hardware and software, and time-intensive personnel training, is confirmed as feasible. Further research will consider employing additional mobile device sensors (e.g., cameras, magnetometer, and barometers) and various communication technologies to improve the accuracy and application range of indoor positioning technology.

## Figures and Tables

**Table 1:** Comparison of Wireless Indoor Positioning.

Technology	Frequency	Accuracy	Advantages	Disadvantages
Bluetooth/ iBeacon	2.4 GHz	cm–m	Low power consumption Small equipment	Software correction required Poor stability
IR	None	cm	High accuracy Low cost	Poor penetration Construction complexity
RFID	125 KHz/ Hundreds of MHz	dm–m	Low cost Short reaction time	Low transmission Poor anti-interference ability
Wi-Fi	2.4 GHz	m	Large-scale positioning High anti-interference ability	High power consumption Low precession
Zigbee	2.4 GHz	m	Low power consumption High efficiency	Slow information transfer Low precession
UWB	3–10 GHz	cm	High precession High security	High cost High power consumption

**Table 2:** Specifications of the experimental mobile phone.

System	Processor	Sensing Device
Android 6.0	Qualcomm^®^ Snapdragon 801, Quad core processor	Accelerometer, Gyroscope, Gravity sensor.

**Table 3:** Basic Attributes of the Fields.

Path	Field 1	Field 2
Type	Closed	Connecting
Length	245 m	92 m
Narrowest width	1.4 m	1.7 m
Number of turns	5	4

**Table 4:** Statistics of PDR positioning (unit: m).

Closure		MAX	MIN	MEAN	RMSE	Relative Accuracy
Field 1	ΔX	196.175	147.390	182.287	182.643	1/1.342
	ΔY	57.313	0.144	24.988	32.085	1/7.638
	2D	201.856	158.189	185.092	185.440	1/1.321
Field 2	ΔX	26.834	14.480	24.067	24.162	1/3.762
	ΔY	70.514	19.545	45.719	47.715	1/1.905
	2D	73.496	26.899	52.019	53.484	1/1.720

**Table 5:** Statistics of PDR correction positioning (unit: m).

Closure		MAX	MIN	MEAN	RMSE	Relative Accuracy
Field 1	ΔX	0.162	0.004	0.086	0.100	1/2444.525
	ΔY	1.042	0.067	0.558	0.649	1/377.532
	2D	1.051	0.072	0.570	0.657	1/373.109
Field 2	ΔX	0.621	0.032	0.244	0.303	1/299.709
	ΔY	0.944	0.032	0.365	0.435	1/209.054
	2D	1.130	0.121	0.455	0.530	1/171.463

**Table 6:** Results of cumulative passing rate.

Type	Field	Criteria (m)	Passing Rate of PDR (%)	Passing Rate of PDR Correction (%)
1/2 Desk level	Field 1	0.500	3.462	79.248
	Field 2	0.500	10.120	77.860
1σ of corrected closure	Field 1	0.657	4.373	85.824
	Field 2	0.530	10.698	79.389
1/2 Aisle width	Field 1	0.708	4.722	87.280
	Field 2	0.845	15.489	89.963
Desk level	Field 1	1.000	5.770	94.688
	Field 2	1.000	16.894	92.937
2σ of corrected closure	Field 1	1.314	7.000	98.272
	Field 2	1.060	17.720	93.846
Aisle width	Field 1	1.416	7.561	98.832
	Field 2	1.690	24.329	98.802
3σ of corrected closure	Field 1	1.971	9.475	99.968
	Field 2	1.590	23.503	98.430
All pass	Field 1	2.4000	10.295	100.000
	Field 2	2.5000	31.475	100.000
